# A Predictive Model of Regional Dementia Prevalence Using Geographic Weighted Regression Analysis

**DOI:** 10.3390/jpm12091388

**Published:** 2022-08-26

**Authors:** Jihye Lim, Jong-Ho Park

**Affiliations:** 1 Department of Health Care and Science, Donga University, Busan 49315, Korea; 2 Division of Health Administration, Gwangju University, Gwangju 61743, Korea

**Keywords:** dementia, hot spot, spatial autocorrelation, geographic weighted regression

## Abstract

Globally, dementia is one of the highest priority public health policy issues. This study was conducted to analyze the spatial distribution pattern of dementia prevalence using geographic weighted regression analysis and to identify preventable risk factors at the regional level of dementia prevalence. For the data to be analyzed, this work used the 2020 regional dementia prevalence index of the Korea Central Dementia Center and the regional health statistics of the Korea Centers for Disease Control and Prevention Agency (KDCA). Spatial autocorrelation analysis, hot spot analysis, and geographic weighted regression analysis were performed to identify regional associations of dementia prevalence, cluster regions with high dementia prevalence, and risk factors for regional dementia prevalence. As a result of the hot spot analysis, the regions corresponding to the hot spots with the high prevalence of dementia were found to be adjacent to each other, such as in Jeonnam, Jeonbuk, and Gyeongbuk, and the regions corresponding to the cold spots with the low prevalence of dementia were adjacent to each other, such as Seoul, Gyeonggi, Incheon, Busan, and Ulsan. The results of geographic weighted regression analysis showed that educational level, walking practice rate, hypertension prevalence, and a low-sodium diet preference were found to be risk factors for the prevalence of dementia. These results suggest that there is a need for a dementia prevalence management strategy to increase the walking practice rate and low-sodium diet preference rate, and decrease the hypertension prevalence, centering on the hot spot area, which is a cluster area with high dementia prevalence. This study is expected to be useful as basic data that can help in prioritizing health policies considering spatial characteristics for community health promotion.

## 1. Introduction

Recently, more attention has been paid to not only personal health but also health on a community level around the world. Macintyre et al. reported that the physical characteristics of the environment, the services provided to support people in their daily lives, the sociocultural characteristics of the community, and the reputation of the region were the factors affecting the health of a community [1]. It has generally been reported that the difference in regional health level is the result of two effects: the composition effect and the context effect [2]. The compositional effect is an interpretation that the level of health in the region differs because the sociodemographic characteristics of the population groups that make up the region are different. The contextual effect is that there are independent factors that affect the health of the region itself which are independent of the characteristic members. These two effects on regional health inequality determine the direction of regional health inequality mitigation policies [3]. The existence of regional factors that can be used to determine health means that it is necessary to take a strategic approach by analyzing the health status and factors of each region.

Dementia is one of the highest priority public health policy issues today. The number of people with dementia worldwide was estimated to be about 46.8 million in 2015, and it is expected to increase to 74.7 million in 2030 and to 131.5 million in 2050 [4]. Dementia refers to a condition in which a person who has been living a normal life has acquired various deficits, including in memory, and various cognitive functions, which severely affect his or her daily life alone. Dementia is not the name of a single disease, but a clinical syndrome that occurs due to the deterioration of mental functioning [5]. Dementia is influenced by non-modifiable age, sex, and genetic factors, but there are also modifiable risk factors [6]. The modifiable factors that can prevent the risk of dementia can be divided into developmental factors, sociopsychological health factors, health behavior factors, and risk factors of cardiovascular disease [6]. These risk factors increase the likelihood of dementia occurring later in life. Previous studies have shown that the known risk factors for dementia include hypertension, obesity, dyslipidemia, diabetes, smoking, depression, and physical activity [7,8]. Research has shown that the risk of developing dementia among patients who have experienced a stroke is approximately twice as high [9]. With the increase in the prevalence of dementia patients, the cost and social burden associated with treating dementia patients is expected to increase rapidly [10]. Therefore, it is urgent to prepare measures to prevent and manage dementia.

As there are regional differences in the prevalence of dementia in Korea [11,12,13], it is necessary to reduce the gap in the prevalence of dementia between regions for effective dementia management. The first law of geography indicates that everything in the world is related, but that spatially, the closer it is, the more closely related it is, and the health level varies due to the spatial characteristics of the region [14,15]. Spatial dependence refers to the existence of a strong correlation with neighboring regions with similar characteristics, although the regions based on administrative units are different [16,17]. To reduce the disparity in the prevalence of dementia between regions, it is necessary to first check that the prevalence of dementia is related between spatially adjacent regions to identify any spatial dependence between regions. If the spatial dependence of the dementia prevalence rate between regions is confirmed, it is necessary to analyze the spatial distribution pattern of the dementia prevalence rate reflecting the spatial characteristics between regions, and to analyze hot spots to find clustered regions with high dementia prevalence rates. Hot spot analysis is a spatial statistical technique that can be used to discriminate spatial patterns of regional health levels and discover clusters in regions with statistically significant high and low health levels [15,16,17]. If such an analysis identifies a dementia prevalence hot spot area, which is a clustered area with a high dementia prevalence rate, it is then necessary to identify preventable dementia risk factors at the regional level and carry out a customized dementia prevention and management project in the hot spots.

Geographically weighted regression analysis is a study method that is used for ecological research at the regional level, and it is a spatial statistical technique that reflects regional spatial characteristics and derives regional health risk factors [18]. Geographically weighted regression analysis not only applies the advantages of the existing regression model in that it uses a regression model, but it also estimates the regression coefficients that are different for each region, so the degree of influence of the risk factors affecting the prevalence of dementia can be confirmed [19]. However, there have been insufficient studies analyzing the spatial dependence of dementia prevalence by region using representative data. Existing studies have conducted a lot of research by approaching individual lifestyle and disease-related risk factors for dementia [8,10,11]. However, as the importance of community health management has been gradually strengthened, the need for research on spatial approaches to diseases using ecological data, rather than individual data, has increased. 

Therefore, in this study, the prevalence and risk factors of dementia among regions in Korea are identified and used as basic data for effective dementia prevention and management projects. The specific goals of this work are as follows:

1. We identify the interregional spatial dependence of dementia prevalence in Korea.
2. We identify clusters in regions with high dementia prevalence through hot spot analysis.
3. We identify risk factors for dementia prevalence in Korea through geographic weighted regression analysis.

## 2. Materials and Methods

### 2.1. Data Collection 

In Korea, public health centers in administrative areas are the institutions that carry out and manage regional health management projects. In this study, 246 administrative regions in Korea, wherein public health centers are installed and operated, were analyzed as the target regions. To confirm the spatial dependence of the prevalence of dementia in Korea and analyze hot spots, we collected the dementia prevalence index from the Korea Central Dementia Center in 2020 (246 regions). To identify risk factors for the prevalence of dementia, the 2020 regional (246 regions) index data of the Korea Disease Control and Prevention Agency (KDCA) regional health statistics were collected. The study was conducted in accordance with the Declaration of Helsinki. Ethical review and approval were waived in this study because it used anonymous public open data and not an individual’s personal data.

### 2.2. Variables and Measures

Dementia prevalence, as a dependent variable, was defined as the proportion (%) of dementia patients among the elderly population over 65 in Korea, according to the definition of dementia prevalence by the Korea Central Dementia Center. The risk factors for the prevalence of dementia, which are independent variables, were selected in consideration of previous studies [6,7,20] and collectible indicators of regional health statistics. The independent variables included education level, obesity rate, prevalence of hypertension, rate of physical activity above moderate level, current smoking rate, prevalence of diabetes, prevalence of depression, prevalence of mild cognitive impairment, proportion of stress recognition, rate of walking practice, high-risk drinking rate of annual drinkers, proportion of the population avoiding skipping breakfast, and low salt preference. The definitions of the variables are presented in Table 1.

### 2.3. Statistical Analysis

The data in this study were analyzed using the IBM SPSS 27.0 (Armonk, NY, USA) and ArcGIS pro 2.6.0 (Esri, Redlands, CA, USA) programs. Descriptive statistical analysis was conducted to understand the prevalence of dementia and the general characteristics of dementia risk factors in the analysis target region. To confirm the spatial dependence of dementia prevalence in Korea, the regional distribution of the dementia prevalence rate was identified through mapping, and a global spatial autocorrelation (Global Moran’s I) analysis was performed. Global spatial autocorrelation (Global Moran’s I) analysis is a representative spatial statistical technique for determining spatial dependence by measuring spatial autocorrelation. Moran’s Index, a spatial autocorrelation index calculated through global spatial autocorrelation analysis, has a range from −1 to 1. If Moran’s Index is 0 or more and close to 1 and statistically significant (*p* < 0.05), then the prevalence of dementia in the target region can be judged to have spatial dependence, which is a spatially strong cluster. 

Hot spot analysis was conducted to identify hot spot regions, which are clusters of regions with high dementia prevalence. In the hot spot analysis, hot spot regions with high statistical values and cold spot regions with low statistical values were classified according to Getis Ord’s G_i_^*^ value. The weight of the distance between regions was analyzed using the K-Nearest Neighbor method by setting the 10 closest regions to the neighboring regions. Geographical weighted regression analysis was conducted to identify risk factors for the prevalence of dementia by region. In the geographic weighted regression analysis, the kernel function type used for the geographic weighting was the adaptive method, which assumed that the locations of the observation cases were irregularly distributed within the study area. To determine the priorities of the management of dementia prevalence by region, the t-test was performed to identify the difference between the risk factor index value and the influence (regression coefficient) of each index of the dementia prevalence rate between hot spot regions and cold spot regions.

## 3. Results

### 3.1. General Characteristics of the Study Regions

Descriptive statistics were conducted to analyze the general characteristics of the study regions (Table 2). As a result of analyzing the average value for each variable, the prevalence were as follows: dementia, 10.86%; low education level, 34.85%; obesity rate, 31.34%; hypertension prevalence, 19.33%; physical activity rate, 21.30%; current smoking rate, 19.66%; diabetes, 8.33%; depression, 2.66%; mild cognitive impairment, 23.01%; stress perception rate, 25.72%; walking practice rate, 37.91%; high-risk drinking rate, 15.75%; proportion of the population avoiding skipping breakfast, 52.32%; and preference rate for low salt, 41.41%.

### 3.2. Spatial Autocorrelation (Global Moran’s I) Analysis of Dementia Prevalence

**Figure 1:**
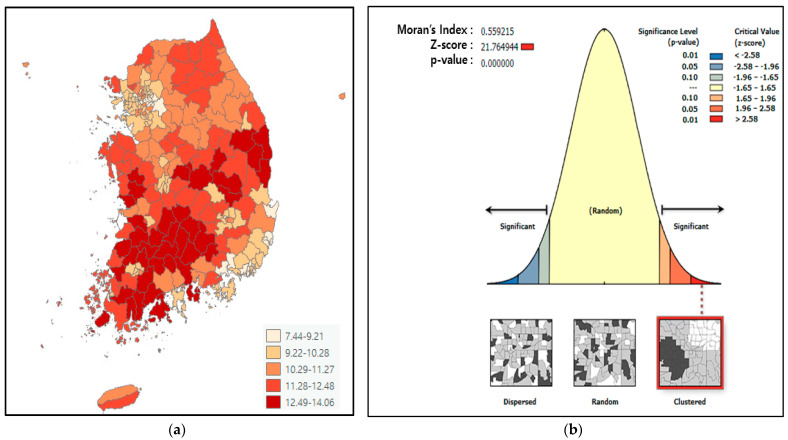
Spatial autocorrelation analysis of dementia prevalence. (**a**) Regional distribution of dementia prevalence; (**b**) global spatial autocorrelation analysis of dementia prevalence.

To determine the regional correlation of dementia prevalence, the regional distribution of dementia prevalence was tested, and spatial autocorrelation analysis was conducted. Mapping the regional distribution of dementia prevalence in Korea, as shown in Figure 1a, indicates that dementia prevalence tends to cluster between regions. Figure 1b shows the result of global spatial autocorrelation analysis. Moran’s Index was 0.559 and Moran’s Index P value was 0.000, indicating statistically significant interregional clustering, thus confirming that the prevalence of dementia in Korea is spatially dependent.

### 3.3. Hot Spot Analysis of Dementia Prevalence

**Figure 2:**
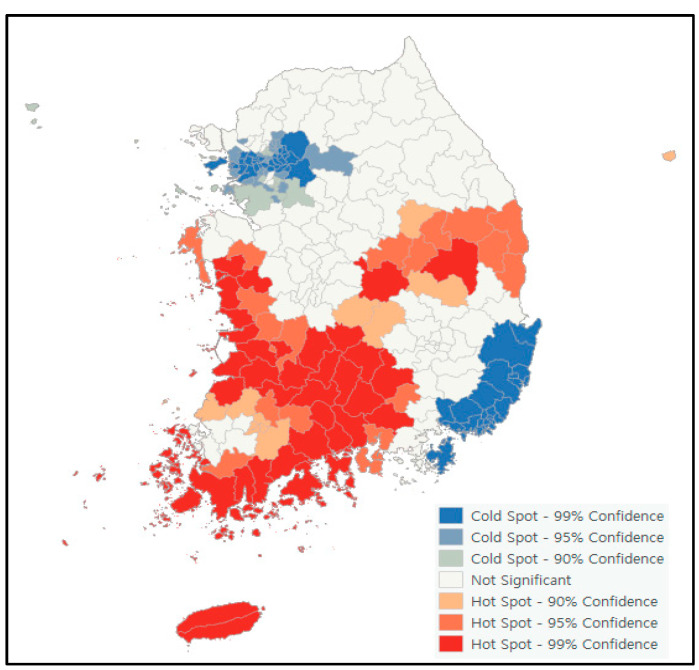
Hot spot distribution of dementia prevalence.

Spatial autocorrelation analysis confirmed that the prevalence of dementia was clustered between regions in a statistically significant manner. Therefore, hot spot analysis was performed to identify hot spot regions, which are clusters of regions with high dementia prevalence. Figure 2 shows the distribution of hot and cold spots. Here, 69 administrative regions in Korea, such as Jeonnam, Jeonbuk, and Gyeongbuk, were found to be hot spots, i.e., clusters of regions with a high prevalence of dementia. Moreover, 88 administrative regions, such as Seoul, Gyeonggi, Incheon, Busan, and Ulsan, were found to be cold spots, i.e., clusters of regions with low dementia prevalence.

### 3.4. Geographically Weighted Regression Analysis in Risk Factors of Dementia Prevalence

Geographically weighted regression (GWR) analysis was conducted to identify risk factors for the prevalence of dementia at the regional level (Table 3). The major risk factors affecting the prevalence of dementia by region in Korea were found to be education level (ratio of level below middle school education), hypertension prevalence, walking practice rate, and low-sodium diet preference rate. Moreover, 246 regional regression models composed of these major risk factors were calculated. The explanatory power of the calculated regression model for 246 regions was found to be distributed from 63.2% to 82.2%. As a result of geographical weighted regression analysis, the influence of each factor was found to differ by region, but based on the average and median values of the regression coefficients, the higher the proportion of those who are less than middle school graduates, the higher the prevalence of hypertension, the lower the walking practice rate; moreover, the lower the preference for a low-sodium diet, the higher the prevalence of dementia.

### 3.5. Priorities of Dementia Prevalence Management

To prioritize the management of dementia prevalence by region, an independent sample t-test was conducted on the difference between the values of risk factor index values for dementia prevalence and the influence (regression coefficient) of each index in hot spot and cold spot regions (Table 4). Regarding the difference in dementia risk factor index values between hot spot and cold spot regions, the ratio of the population with a below middle school education level and the prevalence of hypertension were higher in hot spot areas than in cold spot regions. The walking practice rate and low-sodium diet preference rate were higher in the cold spot regions than in the hot spot regions. As a result of examining the regional differences in the regression coefficients indicating the influence on the prevalence of dementia according to the hot spot regions and cold spot regions, the ratio of a level below a middle school education, the prevalence of hypertension, the walking practice rate, and the low-sodium diet preference rate, the regression coefficients by region were all higher in the hot spot regions than in the cold spot regions based on absolute value. These differences were statistically significant (*p* < 0.05). It was found that dementia prevalence risk factors had a greater influence in the hot spot regions than in the cold spot regions.

## 4. Discussion

For the prevention and management of dementia, it is first necessary to manage the risk factors affecting dementia prior to the diagnosis of dementia. In this regard, studying the effects of regional characteristics on health has great practical implications for public health policy [21]. This is because social interventions may be more successful in changing the environment than they are in changing individuals, and they may be more effective when the unit of intervention is local [22]. Therefore, geographic weighted regression analysis was used in this study to identify preventable risk factors at the regional level of dementia prevalence, and this analysis was conducted to help implement a regionally customized dementia prevention and management project.

As a result of spatial autocorrelation analysis of the dementia prevalence rate, Moran’s index appeared to be 0.371 and Moran’s index p-value was 0.000. The prevalence of dementia is found to be clustered in a statistically significant manner between regions, which can be considered based on Tobler (1970)’s first law of geography, which states that, although everything is related, factors that are closer spatially are more closely related [23,24]. This research method has been verified to be a valid method in previous studies on the identification of risk factors for prevalence at the regional level for hypertension [24,25], diabetes [26], and metabolic syndrome [27]. 

To effectively manage the prevalence of dementia, it is necessary to first find a region with a high prevalence of dementia, and then to conduct intensive management activities centered on such regions. Hot spot analysis is used to identify regions with statistically significant high or low values in data obtained by region, such as disease incidence [28]. As a result of the hot spot analysis, the regions corresponding to the hot spots with a high prevalence of dementia were found to be adjacent to each other, such as Jeonnam, Jeonbuk, and Gyeongbuk, and the regions corresponding to the cold spots with a low prevalence of dementia were also found to be adjacent to each other, such as Seoul, Gyeonggi, Incheon, Busan, and Ulsan. A lot of rural areas were included in the hot spot area, whereas a lot of urban areas were included in the cold spot area. These results are consistent with the results of previous studies [11,12,13] indicating that the prevalence of dementia in rural areas is higher than that in urban areas. In Korea, the prevalence of dementia in the elderly aged 65 and over in 2019 was 10.29% [11], and according to previous studies [29], the prevalence of dementia in rural areas was 1.3~3.2% higher than that in urban areas. The average prevalence of dementia in the hot spot area in this study was 12.26%, and the average prevalence of dementia in the cold spot area was 9.62%. This is considered to be the difference between a cold spot with a lower dementia prevalence among urban areas and a hot spot with a higher dementia prevalence among rural areas. It is necessary to add more facilities and manpower for dementia prevention and management in rural areas, and it is considered to be necessary to prepare a national strategy.

As a result of geographic weighted regression analysis aiming to identify risk factors for the prevalence of preventable dementia at the regional level by reflecting the spatial characteristics of the region, the major risk factors significantly affecting the prevalence of dementia in the region were the education level, the hypertension prevalence, the walking practice rate, and a low-sodium diet preference rate. The prevalence of dementia was found to be high in regions with a high proportion of low educational attainment, which is consistent with the results of previous studies [29,30,31] showing that the lower the educational level, the higher the prevalence of dementia. In previous research [7,32,33], the relative risk of dementia for hypertension was found to be 1.61 and the relative risk of dementia for physical inactivity was found to be 1.82, and these findings are consistent with the results of this study. To effectively manage the prevalence of dementia at the regional level, comprehensive management and monitoring are required for the prevalence of hypertension, walking practice, and preference for a low-sodium diet, which significantly affect the prevalence of dementia. In addition, since low education level has also been shown to affect the prevalence of dementia in the region, it is considered to be necessary to strengthen education and publicity for dementia prevention and early screening in consideration of education level. The walking practice rate can be used not only as the most basic physical activity index indicating the level of local health behavior, but also as a key index representing a good local community to live in as a factor indicating transportation, community safety, and economic vitality [34,35]. Therefore, measures to reduce the regional disparity in walking practice rate not only reduce the risk of dementia but also improve the health and welfare of residents, indicating the need for a strategic approach.

As a result of hot spot analysis and geographic weighted regression analysis to select regional dementia prevalence management priorities, the ratio of the population with a below middle school education level and the prevalence of hypertension were higher in hot spot areas than in cold spot regions. The walking practice rate and low-sodium diet preference rate were higher in the cold spot regions than in the hot spot regions. These results suggest that there is a need for a dementia prevalence management strategy to increase the walking practice rate and low-sodium diet preference rate as well as lower the hypertension prevalence, centering on the hot spot area, which is a cluster area with high dementia prevalence. This also suggests the need for collaborative dementia prevalence management in spatially adjacent hot spot areas for effective management. In Korea, dementia management projects are being actively carried out through collaboration between public institutions, community groups, and community members. In fact, with the support of the state and cooperation with various organizations, early diagnosis and prevention of dementia, and integrated service provision are being carried out, and there has been an improvement in the screening rate for early dementia [32]. In the future, based on the results of this study, strategic action will be needed to alleviate regional differences and shift the paradigm from individual-oriented improvement to regional-oriented improvement.

In this study, using the spatial autocorrelation analysis method validated in previous studies [36,37,38,39,40,41], the inter-regional association of dementia prevalence was identified, and risk factors for dementia prevalence at the regional level were identified as well. It can be said that these findings are methodologically meaningful in that the risk factors for the prevalence of preventable dementia at the regional level reflecting the spatial characteristics of the region were identified using geographic regression analysis. Research using ecological data looks at the relationship between risk factors and health outcomes on a population-by-population basis, and there is an ecological fallacy that differences in population groups fail to infer individual risk-result relationships. However, it is possible to hypothesize that a certain risk can be a potential cause of an outcome through ecological research, and it can be verified through RCT (randomized controlled trials) research. In addition, the advantage of ecological research is that it is inexpensive, and it takes less time, and data can be easily obtained [42]. Nevertheless, this study has the following limitations. First, this study did not consider clinical data variables such as laboratory results that could affect the prevalence of dementia. Second, since community health statistical data has a cross-sectional characteristic, it cannot clearly show the causal relationship between the independent variable and the dependent variable. Third, this study is an ecological study that looks at the relationship between risk factors and health outcomes on a population-group basis and has a limitation in that it cannot collect and analyze individual-level dementia risk factors. In the future, in-depth studies necessary for alleviating regional health imbalances should be conducted to supplement these limitations.

## 5. Conclusions

In this study, the spatial distribution pattern of the dementia prevalence rate reflecting spatial characteristics between regions was identified, and the characteristics of clustered regions with high dementia prevalence rates were identified as well. The results of this study showed that the regions could be classified into cluster areas with a high prevalence of dementia (hot spots) and cluster areas with a low prevalence of dementia (cold spots), which also means that there is health inequality between regions. Factors affecting health are not evenly distributed across regions. The socio-demographic characteristics, physical environment, and awareness and participation of community residents are all community factors that affect health [43]. Therefore, identifying and intervening at the national level with community factors that determine health may be a major strategy for controlling health inequality. Based on these research results, in planning policies for dementia prevention management and health promotion, it will be necessary to effectively plan an action strategy that considers regional group-based risk factors in consideration of spatial characteristics. It is expected that the results of this study will be useful as basic data that can help prioritize health policies considering spatial characteristics for community health promotion. In the future, in consideration of the limitations of the study, research should be conducted that considers the root cause of spatial clustering and various factors.

## Figures and Tables

**Table 1:** Variable definitions.

Variables	Definition	Source (year)
Dementia prevalence	The proportion of the dementia population aged 65 and over among the population aged 65 and over (%)	Korea Central Dementia Center (2020)
Prevalence of mild cognitive impairment	The proportion of those with mild cognitive impairment aged 65 and over among the population aged 65 and over (%)	
Education level	The proportion of those with an education level below middle school education among the population aged 19 and over (%)	Community HealthSurvey (2020), KDCA ^1^
Obesity prevalence	The proportion of the population with a body mass index of 25 and over (%)	
Hypertension prevalence	The proportion of the hypertension population aged 30 and over (diagnosed) (%)	
Diabetes prevalence	The proportion of the diabetes population aged 30 and over (diagnosed) (%)	
Depression prevalence	The proportion of the population with a total score of 10 or over on the Patient Health Questionnaire-9 (PHQ-9) (%)	
Current smoking	The proportion of the population who smoked more than five packs (100 cigarettes) in their lifetime, and who currently smoke (%)	
Moderate-to-high physical activity	The proportion of the population who engaged in high physical activity for at least 20 minutes a day, over 3 days in a recent week, or moderate physical activity, at least 30 minutes, over 5 days in a recent week (%)	
Stress recognition	The proportion of the population who feel ‘very stressful’ or ‘stressful’ in daily life (%)	
Walking practice	The proportion of the population who practiced walking at least 30 minutes a day, over 5 days in a recent week (%)	
High-risk drinking	The proportion of the population who drink alcohol more than twice a week; over seven glasses (or five cans of beer) for men, over five glasses (or three cans of beer) for women at once (%)	
Avoiding skipping breakfast	The proportion of the population who only had breakfast five or more times a week in the past year (%)	
Low-sodium diet preference	The proportion of the population who usually prefer a low-sodium diet (%)	Community Health Survey (2019) *, KDCA ^1^

^1^ KDCA: Korea Disease Control and Prevention Agency; * The low salt preference rate was not surveyed in 2020, so 2019 data were used.

**Table 2:** General characteristics of study regions (246 regions).

Variables	Min	Max	Average	SD ^1^	EQ ^2^	CV ^3^
Dementia prevalence	7.44	14.06	10.86	1.40	6.62	0.13
Prevalence of mild cognitive impairment	21.01	24.85	23.01	0.78	3.84	0.03
Education level	5.38	66.32	34.85	15.69	60.94	0.45
Obesity prevalence	20.10	43.50	31.34	3.45	23.40	0.11
Hypertension prevalence	14.10	26.80	19.33	2.31	12.70	0.12
Diabetes prevalence	4.30	13.30	8.33	1.41	9.00	0.17
Depression prevalence	0.00	6.40	2.66	1.34	6.40	0.51
Current smoking	10.10	29.30	19.66	3.13	19.20	0.16
Moderate-to-high physical activity	7.80	62.40	21.30	7.22	54.60	0.34
Stress recognition	6.20	36.10	25.72	4.86	29.90	0.19
Walking practice	14.20	82.00	37.91	10.92	67.80	0.29
High-risk drinking	6.50	29.20	15.75	3.97	22.70	0.25
Avoiding skipping breakfast	37.30	67.70	52.32	5.53	30.40	0.11
Low-sodium diet preference	23.50	62.40	41.41	6.04	38.90	0.15

^1^ SD: standard deviation, ^2^ EQ: max-min, ^3^ CV: coefficient of variation; EQ and CV were presented to measure regional variation.

**Table 3:** Risk factors affecting dementia prevalence (GWR).

Variables	Regression Coefficient			
	Average	Median	Min	Max
Education level (level below middle school education)	0.069	0.068	0.057	0.086
Hypertension prevalence	0.023	0.024	0.003	0.041
Walking practice	–0.018	–0.017	–0.022	–0.002
Low-sodium diet preference	–0.012	–0.013	–0.015	0.006
Regional coefficient	0.732	0.740	0.632	0.822
R-square/Adj R-square	0.743/0.727			

**Table 4:** Differences in risk factors for dementia prevalence in hot spot and cold spot regions.

Variables		Hot Spot Region			Cold Spot Region			*p*
		N ^1^	Average	SD ^2^	N ^1^	Average	SD ^2^	
Indicator value	Dementia prevalence	69	12.264	1.117	88	9.623	0.739	0.000
	Education level	69	49.955	12.011	88	22.177	7.999	0.000
	Hypertension prevalence	69	20.961	2.241	88	18.945	2.122	0.005
	Walking practice	69	31.981	9.711	88	44.780	8.583	0.000
	Low-sodium diet preference	69	39.106	7.294	88	42.258	4.321	0.002
Regression coefficient	Education level	69	0.074	0.005	88	0.068	0.010	0.000
	Hypertension prevalence	69	0.032	0.007	88	0.017	0.006	0.000
	Walking practice	69	–0.019	0.001	88	–0.016	0.002	0.000
	Low-sodium diet preference	69	–0.014	0.001	88	–0.008	0.005	0.000

^1^ N: number of regional units, ^2^ SD: standard deviation.

## Data Availability

Restrictions apply to the availability of these data. Data were obtained from the Korea Disease Control and Prevention Agency (KDCA) and are available from https://chs.kdca.go.kr/chs/recsRoom/dataBaseMain.do (accessed on 2 March 2022).
